# Editorial: Mitochondrial quality control in cardiovascular diseases

**DOI:** 10.3389/fcvm.2023.1243895

**Published:** 2023-07-12

**Authors:** Miao Zhang, Rongjun Zou, Ge Wang, Xiaoping Fan

**Affiliations:** ^1^ Department of Cardiovascular Surgery, Guangdong Provincial Hospital of Chinese Medicine, The Second Affiliated Hospital of Guangzhou University of Chinese Medicine, Guangzhou, China; ^2^ State Key Laboratory of Dampness Syndrome of Chinese Medicine, The Second Affiliated Hospital of Guangzhou University of Chinese Medicine, Guangzhou, China; ^3^ The Second Clinical College of Guangzhou, University of Chinese Medicine, Guangzhou, China; ^4^ Department of Cardiovascular Surgery, Guangdong Cardiovascular Institute, Guangdong Provincial Key Laboratory of South China Structural Heart Disease, Guangdong Academy of Medical Sciences, Guangdong General Hospital, Guangzhou, China

**Keywords:** mitochondrial dynamics, mitophagy, mitochondrial biogenesis, cardiovascular diseases, traditional Chinese medicine

**Editorial on the Research Topic** Regulation network of mitochondrial structural and functional disorders in cardiovascular diseases

Myocardial mitochondria are the primary site of energy metabolism in the heart. Thus, maintaining the quality of mitochondria plays a vital role in cardiovascular diseases. Mitochondrial quality control (MQC) mainly encompasses the regulation of mitochondrial dynamics, autophagy, and biogenesis. The pathogenic mechanisms of abnormal mitochondrial features in cardiovascular diseases remain complex, but understanding them may provide promising potential targets for the discovery of new diagnostic and therapeutic approaches for cardiovascular diseases. This editorial is a collection of original research and review articles to summarize our current understanding of the molecular mechanisms of mitochondrial dysfunction in cardiovascular diseases and explore future research directions in this area.

Oxidative stress is the primary cause of mitochondrial dysfunction. While small quantities of reactive oxygen species (ROS) do not pose harm to life activities, excessive accumulation of ROS serves as a risk factor for oxidative stress. Oxidative stress is characterized by the inhibition of mitochondrial respiratory enzyme activity and the slowing of electron transfer within the respiratory chain. Consequently, mitochondrial dysfunction occurs, which, in turn, leads to the accumulation of more reactive oxygen species in the mitochondria, causing greater damage. A review by Wang et al. on mitochondrial oxidative stress in cardiac microvascular ischemia/reperfusion (I/R) injury elucidated that elevated levels of mitochondrial reactive oxygen species (mROS) in endothelial cells can downregulate the expression of endothelial nitric oxide synthase (eNOS), leading to reduced vasodilation. This can result in vasoconstriction, limiting blood reperfusion to the heart and exacerbating cardiac injury (1).

Mitochondrial morphological changes make them more adaptable to cellular functions. Mitochondrial dynamics refers to the dynamic balance between mitochondrial fission and fusion. Mitochondrial fission is mainly regulated by dynamin-related protein 1 (Drp1), mitochondrial fission protein 1 (FIS1), mitochondrial fission factor (MFF), and mitochondrial dynamics proteins of 49/51 KDa (MID49/51). Meanwhile, mitochondrial fusion is mainly regulated by mitofusin 1/2 (Mfn1/2) and optic atrophy 1 (OPA1). A study on I/R demonstrated that myocardial I/R injury substantially downregulated dual specificity protein phosphatase 1 (DUSP1), which led to the phosphorylation of c-Jun N-terminal kinase (JNK); activated JNK increased MFF expression, triggering lethal mitochondrial fission that resulted in scar expansion, cardiac dysfunction, and cell death (2).

Mitochondrial autophagy is an intracellular mechanism that selectively degrades functionally abnormal mitochondria. When stimulated by external events, autophagic precursors specifically envelop damaged mitochondria and fuse with lysosomes to form autolysosomes that degrade those enveloped mitochondria and replenish the components required for mitochondrial production. Mitochondrial autophagy is a double-edged sword. While moderate upregulation confers protection, excessive mitochondrial autophagy causes oxidative stress damage in cardiomyocytes. PTEN-induced kinase 1 (PINK1) is a mitochondrial serine/threonine kinase primarily degraded by mitochondrial proteases under physiological conditions. If mitochondrial damage occurs, the mitochondrial membrane potential is depolarized and the mitochondrial protease activity is reduced, making it possible for PINK1 to accumulate on the outer mitochondrial membrane and recruit Parkin from the cytoplasm to the mitochondria. Once localized to the mitochondria, Parkin can attract the autophagy receptor p62 to bind to the autophagosome-resident protein LC3, which then facilitates the delivery of ubiquitinated mitochondria to the autophagosomes. In addition to PINK1/Parkin-mediated mitochondrial autophagy, the receptor-dependent pathway induced by mitochondrial autophagy includes FUN14 domain containing 1 (FUNDC1). FUNDC1 contains a sequence that interacts with the LC3, which is essential for the attachment of damaged mitochondria to autophagosomes. In cases where mitochondrial damage occurs, FUNDC1 is dephosphorylated, mostly at sites Ser13, Ser16, and Tyr17, which boosts FUNDC1-LC3 binding and therefore mitochondrial autophagy. Under physiological conditions, DNA-dependent protein kinase catalytic subunit (DNA-PKcs) can sense DNA damage and initiate DNA double-strand repair; however, excessive DNA-PKcs activation can be malignant. Excessive DNA-PKcs activation phosphorylates p53, activates Bax translocation from the cytosol to the outer mitochondrial membrane, and enhances the mitochondrial membrane's permeability, thus inducing apoptosis (3). In response to cardiac I/R injury, Bax inhibitor-1 (BI-1) shows little change at the transcription level, while its protein expression is downregulated due to DNA-PKcs activation. Our study demonstrates that BI-1 exerts endogenous protective effects in cardiac reperfusion injury by repressing fission-related factors, reversing fusion-related elements, and upregulating mitochondrial autophagy-related proteins like FUNDC1 and Parkin (4).

Mitochondrial biogenesis refers to the process of synthesizing and proliferating functional mitochondria, which helps maintain the overall functionality of mitochondria and cellular energy metabolism. Mitochondrial biogenesis is critically regulated by peroxisome proliferator-activated receptor-γ coactivator 1α (PGC-1α), which modulates nuclear-encoded nuclear respiratory factors to regulate nuclear gene expression in the mitochondrial oxidative phosphorylation system. Our previous study on diabetic cardiomyopathy demonstrated that in streptozotocin (STZ)-induced diabetic mice, PGAM family member 5 (PGAM5) protein expression was upregulated, which was associated with impaired cardiac function. PGAM5 directly binds to prohibitin 2 (PHB2) located on the mitochondrial inner membrane, resulting in its dephosphorylation at site Ser91. Dephosphorylated PHB2 disrupts the MQC system in cardiomyocytes, impairing mitochondrial dynamics, mitochondrial autophagy, and mitochondrial biogenesis, which leads to myocardial dysfunction in diabetic mice. Conversely, normal levels of Drp1/FIS1 and Mfn2/OPA1, restored expression of autophagy-related proteins Parkin, Beclin1, and autophagy-related protein 5 (ATG5), and increased mRNA expression of mitotic PGC-1α, nuclear respiratory factor 2 (Nrf2), and translocation-associated membrane protein (TRAM) were observed in the heart tissue of STZ-treated PGAM5^CKO^ mice (5).

Several studies have shown that the therapeutic mechanisms of some compounds, monotherapies, and monomer components of traditional Chinese medicines for cardiovascular diseases may be linked to mitochondria. For example, ginsenoside Rg5 can improve isoproterenol-induced myocardial ischemia by inhibiting mitochondrial aggregation and reducing mitochondrial fission, which is mediated by Drp1 and involves the interaction between mitochondria and endoplasmic reticulum; berberine can activate the AMPK signalling pathway to boost mitochondrial biogenesis, restore autophagic flow, and reduce high glucose-induced cardiomyocyte injury; salidroside can regulate the impaired energy metabolism of mitochondria in cardiomyocytes by improving mitochondrial respiratory function and increasing ATP synthesis (6). Currently, there are numerous compounds and monomer components demonstrating favorable therapeutic effects on cardiovascular diseases. Chen's research group found that aloe-emodin and puerarin can inhibit the activation of Nlrp3 inflammasomes, restore endothelial gap junction proteins, and enhance the permeability of vascular endothelial cells (7, 8). Additionally, numerous Chinese herbal compounds and monomers have proven to be effective in clinical practice, including Qili Qiangxin capsules, Lingguizhugan decoction, and andrographolide for the treatment of chronic heart failure; Qishen granules for the treatment of myocardial infarction; Shexiang Tongxin dropping pills, Yixin Jiedu formula, and compound Danshen dripping pills for the improvement of myocardial ischemia; and Sanhuang Xiexin decoction and Longshengzhi capsules for the intervention of atherosclerosis. However, it is still necessary to explore whether the above-mentioned compounds and monomer components impact the maintenance of mitochondrial homeostasis.

**Figure 1:**
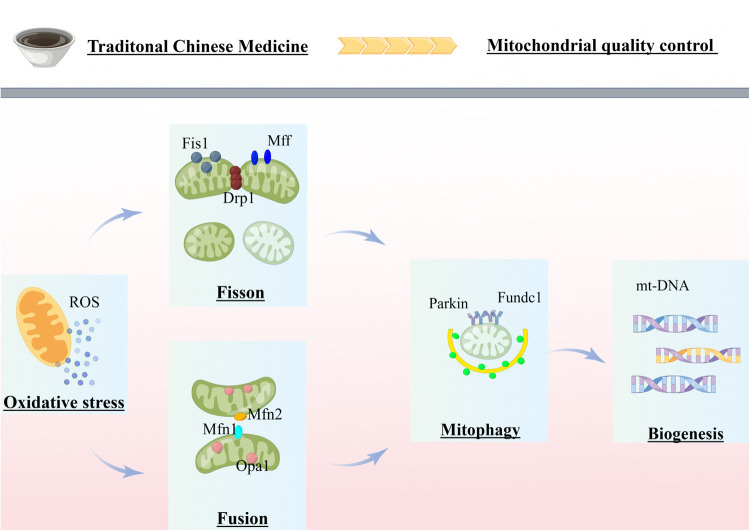
Review of mitochondrial quality control in cardiovascular disease. The figure was completed on the website of Figdraw. MQC coordinates various processes (fission, fusion, mitophagy and biogenesis) to ensure cellular homeostasis. Mitochondrial dysfunction, exacerbated by failing quality-control processes, is believed to be a major mechanism of cardiovascular disease. Chinese herbal compounds and monomers can treat cardiovascular diseases by inhibiting mitochondrial fission, promoting mitochondrial fusion, and moderately activating mitochondrial autophagy by acting on several potential targets of MQC.

In summary, Figure 1 provides a comprehensive overview of the research advancements and potential research directions pursued by our team in the field of mitochondrial quality control related to cardiovascular diseases. In response to stress or injury, mitochondrial metabolic adaptations are activated to eliminate excessive ROS and maintain adequate energy supply. If these metabolic adaptations fail to repair mitochondrial stress, dysregulated mitochondria can fuse with healthy mitochondria and restore homeostasis. In case of accumulated mitochondrial damage, damaged mitochondria can split into several fragments, which are then degraded via mitochondrial autophagy. The activation of mitochondrial autophagy reduces the number of mitochondria, leading to the activation of biogenesis to generate new mitochondria. Mitochondrial fission coordinates with fusion, autophagy, and biogenesis to maximize the preservation of healthy mitochondria while expelling irreversibly damaged mitochondria, achieving the dual regulation of mitochondrial quantity and quality. Thus, MQC is critical in the pathological process of cardiovascular diseases. Currently, there are numerous traditional Chinese medicines and compounds with favorable therapeutic effects for cardiovascular diseases. Further research is necessary to deeply explore medicinal extracts and monomer components that can regulate mitochondrial dysfunction in cardiomyocytes, providing theoretical support for clinical medication.
